# Biomarkers for Acute Respiratory Distress syndrome and prospects for personalised medicine

**DOI:** 10.1186/s12950-018-0202-y

**Published:** 2019-01-15

**Authors:** Savino Spadaro, Mirae Park, Cecilia Turrini, Tanushree Tunstall, Ryan Thwaites, Tommaso Mauri, Riccardo Ragazzi, Paolo Ruggeri, Trevor T. Hansel, Gaetano Caramori, Carlo Alberto Volta

**Affiliations:** 1 Department of Morphology, Surgery and Experimental Medicine, Intensive Care Section, University of Ferrara, 44121 Ferrara, Italy; 2 Faculty of Medicine, National Heart and Lung Institute, Imperial College London, London, UK; 3 Department of Anesthesia, Critical Care and Emergency, Fondazione IRCCS Ca’ Granda Ospedale Maggiore Policlinico, Milan, Italy; 4 Unità Operativa Complessa di Pneumologia, Dipartimento di Scienze Biomediche, Odontoiatriche e delle Immagini Morfologiche e Funzionali (BIOMORF), Università di Messina, Messina, Italy

**Keywords:** Acute respiratory distress syndrome, Biomarkers, Inflammatory

## Abstract

Acute lung injury (ALI) affects over 10% of patients hospitalised in critical care, with acute respiratory distress syndrome (ARDS) being the most severe form of ALI and having a mortality rate in the region of 40%. There has been slow but incremental progress in identification of biomarkers that contribute to the pathophysiology of ARDS, have utility in diagnosis and monitoring, and that are potential therapeutic targets (Calfee CS, Delucchi K, Parsons PE, Thompson BT, Ware LB, Matthay MA, Thompson T, Ware LB, Matthay MA, Lancet Respir Med 2014, 2:611–-620). However, a major issue is that ARDS is such a heterogeneous, multi-factorial, end-stage condition that the strategies for “lumping and splitting” are critical (Prescott HC, Calfee CS, Thompson BT, Angus DC, Liu VX, Am J Respir Crit Care Med 2016, 194:147–-155). Nevertheless, sequencing of the human genome, the availability of improved methods for analysis of transcription to mRNA (gene expression), and development of sensitive immunoassays has allowed the application of network biology to ARDS, with these biomarkers offering potential for personalised or precision medicine (Sweeney TE, Khatri P, Toward precision medicine Crit Care Med; 2017 45:934-939).

Biomarker panels have potential applications in molecular phenotyping for identifying patients at risk of developing ARDS, diagnosis of ARDS, risk stratification and monitoring. Two subphenotypes of ARDS have been identified on the basis of blood biomarkers: hypo-inflammatory and hyper-inflammatory. The hyper-inflammatory subphenotype is associated with shock, metabolic acidosis and worst clinical outcomes. Biomarkers of particular interest have included interleukins (IL-6 and IL-8), interferon gamma (IFN-γ), surfactant proteins (SPD and SPB), von Willebrand factor antigen, angiopoietin 1/2 and plasminogen activator inhibitor-1 (PAI-1). In terms of gene expression (mRNA) in blood there have been found to be increases in neutrophil-related genes in sepsis-induced and influenza-induced ARDS, but whole blood expression does not give a robust diagnostic test for ARDS.

Despite improvements in management of ARDS on the critical care unit, this complex disease continues to be a major life-threatening event. Clinical trials of β_2_-agonists, statins, surfactants and keratinocyte growth factor (KGF) have been disappointing. In addition, monoclonal antibodies (anti-TNF) and TNFR fusion protein have also been unconvincing. However, there have been major advances in methods of mechanical ventilation, a neuromuscular blocker (cisatracurium besilate) has shown some benefit, and stem cell therapy is being developed. In the future, by understanding the role of biomarkers in the pathophysiology of ARDS and lung injury, it is hoped that this will provide rational therapeutic targets and ultimately improve clinical care (Seymour CW, Gomez H, Chang CH, Clermont G, Kellum JA, Kennedy J, Yende S, Angus DC, Crit Care 2017, 21:257).

## Introduction

### Definition of ARDS

The definition and the criteria for the diagnosis of ARDS have changed many times during the years. The first description of an ARDS-like syndrome appeared in 1967 grouping together patients with acute respiratory failure associated with dyspnea, tachypnea, cyanosis that is refractory to oxygen therapy, decreased lung compliance, and diffuse alveolar infiltrates evident on the chest radiograph [1].

The 1994 American–European Consensus Conference (AECC) defined ARDS according to the presence of all the following clinical criteria: a) recent onset of symptoms after a known risk factor, b) severe hypoxemia defined by a PaO_2_/FiO_2_ ratio less than 200 mmHg, c) bilateral infiltrates on chest radiograph, d) absence of cardiogenic pulmonary edema [2]. The AECC coined the term Acute Lung Injury (ALI) to facilitate diagnosing patients earlier in the course of their ARDS and identify patients who have a milder form of acute hypoxemic respiratory failure than ARDS.

The Berlin Clinical Classification of ARDS was established to classify patients according to their disease. The current working definition of proposed three mutually exclusive categories (mild, moderate, severe) of ARDS severity (Table 1). These are based on degree of hypoxemia [3]. In the revised Berlin definition, the term ARDS was redefined as a broader concept including a milder condition of lung injury; therefore, it became equivalent to acute lung injury (ALI), which was the previous AECC definition.

**Table 1:** Current definition of ARDS: the Berlin definition [3]

*Timing*	Within 1 week of a known clinical insult or new or worsening respiratory symptoms	
*Chest imaging (chest radiograph or computed tomography scan)*	Bilateral opacities; not fully explained by effusions, lobar/lung collapse, or nodules	
*Origin of edema*	Respiratory failure not fully explained by cardiac failure or fluid overload Need objective assessment (e.g., echocardiography) to exclude hydrostatic edema if no risk factor present	
*Oxygenation*	Mild	200 mmHg < PaO_2_/FIO_2_ ≤ 300 mmHg with PEEP or CPAP ≥5 cmH_2_O
	Moderate	100 mmHg < PaO_2_/FIO_2_ ≤ 200 mmHg with PEEP ≥5 cmH_2_O
	Severe	PaO_2_/FIO_2_ ≤ 100 mmHg with PEEP ≥5 cmH_2_O

### Epidemiology of ARDS

The acute respiratory distress syndrome (ARDS) represents a major cause of death in the critical care units worldwide, with mortality rates around 40% [4] even with the latest advances in its treatment [4, 5]. In recent prospective study carried out in 459 ICUs in 50 countries in 5 continents, ARDS appeared to be underrecognized and undertreated, with some geographic variation and with confirmed high mortality [6]. In this multicenter study ARDS has shown to represent 10.4% of total ICU admission and 23.4% of all patients requiring mechanical ventilation. The prevalence of mild ARDS was 30.0%, moderate, 46.6% and severe, 23.4%. Overall, unadjusted ICU and hospital mortality was 35.3 and 40.0%, respectively and both augmented with increased ARDS severity [6].

### Aetiology of ARDS

In the Berlin definition ARDS has been defined by exposure to a known clinical insult or worsening of respiratory symptoms within 7 days. Although the exact cause of ARDS is not always determinable, it is important to identify the risk factors associated. Generally, risk factors are divided into direct and indirect causes of lung injury (Table 2). The most common causes of indirect ARDS are pneumonia and sepsis. However, these are not the only drivers that lead to the development of ARDS but other unknown factors play a role in the pathogenesis. Among them genetic factors may be involved although no single gene polymorphism has shown significant predisposition to ARDS. Moreover, virulence factors and environmental ones (such as exposition to injurious mechanical ventilation) may contribute to the progression of the disease to ARDS [7, 8]. An ideal biomarker should provide information for identification of patients at risk for ARDS and with different ARDS phenotypes during the progression of lung injury. Indeed several candidate biomarkers for ARDS that have been investigated in blood, pulmonary edema fluid, and exhaled air, but currently they are not reliable enough for clinical use. A combination of biomarkers could help distinguish patients with direct lung injury from those with an indirect mechanism of lung injury, thus helping in the diagnosis and identification of patients that may benefit from different therapeutic strategies (Table 3).

**Table 2:** Risk factors commonly associated with ARDS

*Direct lung injury*	*Indirect lung injury*
Pneumonia	Sepsis
Aspiration of gastric contents	Multiple trauma
Pulmonary contusion	Cardiopulmonary bypass
Near drowning	Acute pancreatitis
Inhalation injury	Drug overdose
Reperfusion pulmonary edema	Transfusion of blood products

**Table 3:** Biomarkers of ARDS [13, 15, 92, 93]

Pathway	Biomarkers	
*Epithelial*	RAGE	
	SP-D	
	KL-6	
	CC16	
	KGF	
*Endothelial*	Ang-1/2	
	vWF	
	VEGF	
*Inflammatory*	Pro-inflammatory	IL-1β
		IL-6
		TNFα
		IL-8
		IL-18
	Anti-inflammatory	IL-1RA
		sTNF-RI/II
		IL-10
*Coagulation and fibrinolysis*	PAI-1	

## Pathology

The pathophysiology of ALI/ARDS is complex and remains incompletely understood.

Interestingly all the above definitions of ARDS do not include the presence of an inflammatory process of the lower airways. Despite this, ARDS is currently considered to represent a stereotypic response to many different injuries all evolving through a number of different phases: alveolar and capillary damage to lung resolution with or without a fibro-proliferative phase.

Furthermore, ARDS is not characterized by a specific clinic-pathological disease but includes a heterogeneous list of clinic-pathological entities, usually showing diffuse alveolar damage (DAD) with severe widespread damage to the alveolo-capillary unit [9].

### Early and late phases of lung injury

**Fig. 1:**
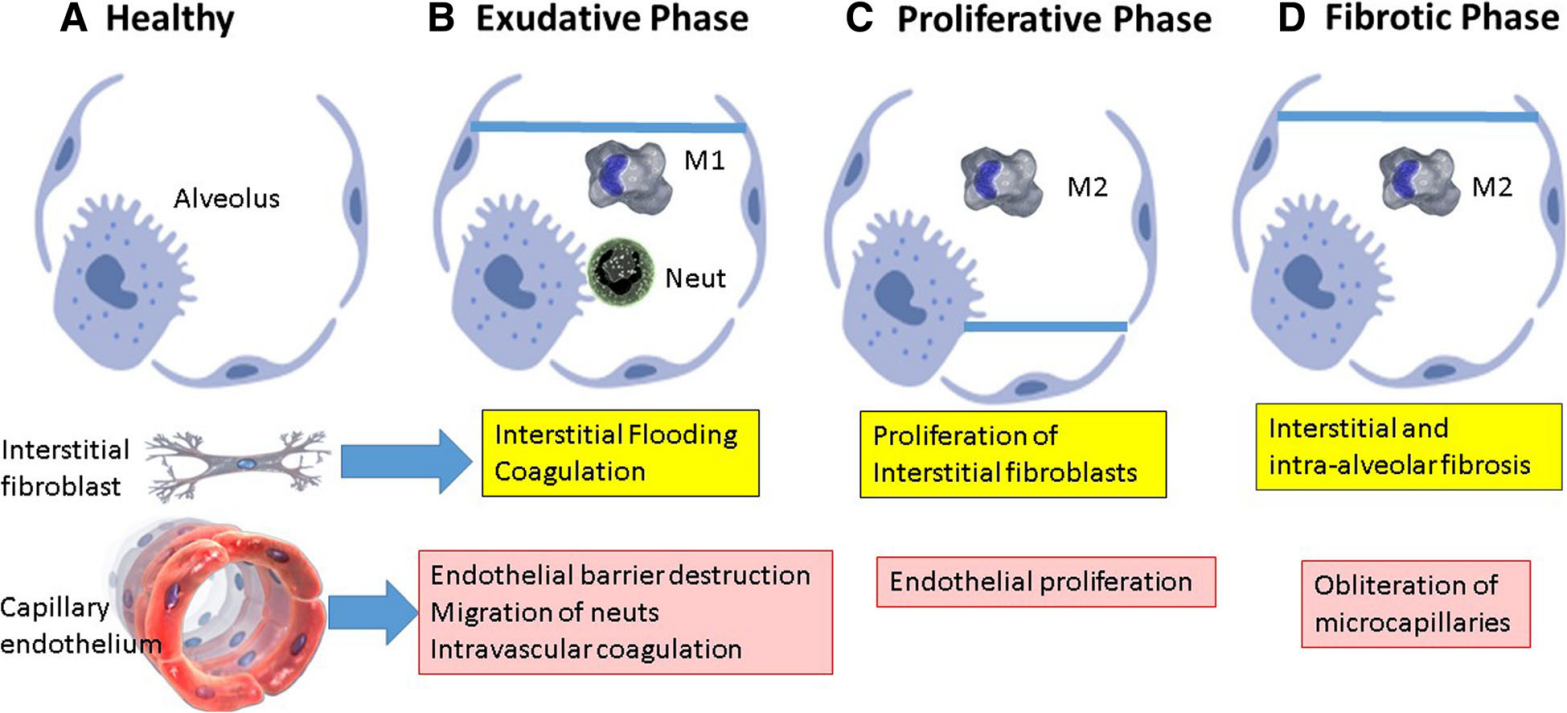
Immunopathology and biomarkers of ARDS. Diagram illustrate the key cells and molecules involved in the pathophysiology of ARDS

Pathologically and clinically, ARDS can be divided into early and late phases of lung injury, corresponding to exudative and fibroproliferative phases [10] (Fig. 1). This involves endothelial and epithelial damage, the inflammatory cascade, and increased vascular permeability. There are associated alterations in lung matrix, activation of coagulation and fibrosis pathways, with cell proliferation and apoptosis.

A. Early phase: In the early phase (first few hours or days), there is widespread neutrophilic alveolitis with disruption of the alveolar epithelial and endothelial barriers, while leads to the formation of protein-rich edema in the interstitium and alveolar spaces [8]. Microscopically, lungs in the early stages show diffuse alveolar damage (DAD) with alveolar flooding by proteinaceous fluid, neutrophil influx into the alveolar space, loss of alveolar epithelial cells, deposition of hyaline membranes on the denuded basement membrane and formation of microthrombi [11]. Inflammatory cell infiltration of the lung interstitium may also be seen. The alveolar flooding occurs as a result of injury to the alveolar-capillary barrier and is a major determinant of the hypoxemia and altered lung mechanics that characterize early ALI/ARDS. Injury to the alveolar epithelium is a prominent feature histologically with loss of alveolar epithelial barrier integrity and extensive necrosis of alveolar epithelial type I cells. Endothelial injury allows leakage of plasma from the capillaries into the interstitium and airspace. The mechanism by which the microvascular endothelium and alveolar epithelium are injured are probably multiple and may vary depending on the inciting event.

B. Late Phase: Disordered healing and proliferation of fibrous tissue dominate the late phase of ARDS. The scenario evolves to a fibro-proliferative process that fills the airspaces with granulation tissue containing proliferating alveolar type II cells, as well as new blood vessels and extracellular matrix rich in collagen and fibrin. Type II alveolar cell, fibroblast and myofibroblasts proliferate in this phase, which can occur as early as 7 to 10 days after initial injury. This stage has traditionally been described as being followed by a fibrotic phase, essentially emphasizing the appearance of pulmonary fibrosis in a subset of patients with irreversible lung fibrosis [11]. Recent observations have suggested that the areas of fibrosis may develop sooner than previously appreciated. Elevated levels of N-terminal procollagen peptide III, thought to represent collagen synthesis, can be detected in bronchoalveolar lavage fluid of patients with ARDS as early as 24 h into the course of the illness [12]. This observation have led some investigators to hypothesize that fibroproliferation may be initiated simultaneously with inflammatory lung injury [12].

## Biomarkers

**Fig. 2:**
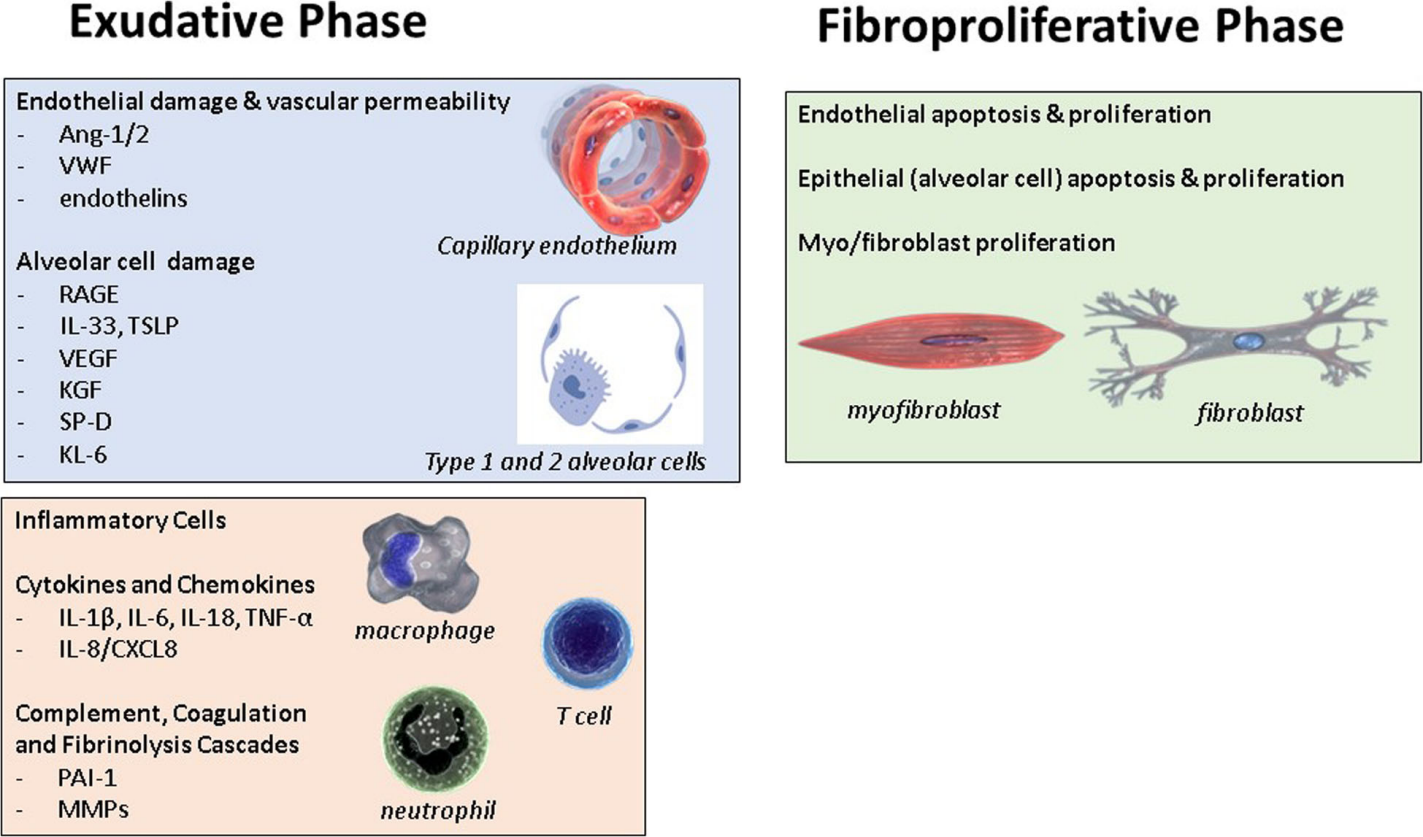
Diagram to illustrate the Key Cells and Molecules involved in the Immune. Pathophysiology of ARDS: with an emphasis on biomarkers that have been measured in plasma (based on the work of the Carolyn Calfee group)

In ARDS biomarkers have promise in diagnosis and stratification, assessment of prognosis and to evaluate response to therapy [13, 14] (Fig. 2). Sequencing of the human genome, the availability of improved methods for analysis of transcription to mRNA (gene expression), and development of sensitive immunoassays has allowed the application of network biology to ARDS, with these biomarkers offering potential for personalised or precision medicine [15].

### Epithelial markers

Respiratory epithelium markers include surfactant proteins (SP); Krebs von den Lungen-6 (KL-6) protein, vascular endothelial growth factor (VEGF) and soluble receptor for advanced glycation end-products (sRAGE)

- Surfactant proteins (SP) are generally increased in ARDS, and SP-B can cross damaged alveolocapillary membranes [16, 17]. Blood SP-D levels been shown to correlate with ARDS mortality [18, 19].
- KL-6 levels have been correlated with ARDS mortality as opposed to ARDS development [20]. In 2014 a meta-analysis of plasma biomarkers for ARDS analysed 54 studies found KL-6, lactate dehydrogenase, sRAGE, and von Willebrand factor were associated with ARDS diagnosis in at risk populations [21].
- VEGF and keratinocyte growth factor were shown to correlate with severity of illness and reflect patient outcome [22, 23].
- RAGE is highly expressed in lung epithelium [24], and especially expressed on alveolar type 1 epithelial cells [25]. The use as a marker has been questionable but some studies have shown higher levels of RAGE were associated with impaired alveolar fluid clearance in patients with ARDS hence the severity of lung epithelial injury [26]. RAGE plasma levels in patients with severe ARDS correlated with mortality in patients ventilated with high tidal volumes [27]. In a meta-analysis sRAGE was found to be useful in ARDS diagnosis in a high risk population, but not associated with mortality [21], although other studies have shown no association [28, 29].

### Endothelial markers

Endothelial markers include angiopoietin-2 (Ang-2) and markers of endothelial dysfunction [30]. Elevated levels of Ang-2 in both ARDS and at risk patients are predictive of mortality [29, 31, 32] and there is a correlation between Ang-2 levels and ARDS development in trauma patients [33]. In addition, for VWF here seems to be a correlation with mortality in ARDS [18, 34, 35].

### Inflammatory cytokines

Levels of the inflammatory cytokines IL-1β and TNF-α are more useful as markers of sepsis severity rather than for ARDS [36]. Other pro-inflammatory cytokines include IL-8, which has been shown in predicting the outcome of ARDS [18, 34]. IL-18 has been noted to be increased in patients with ARDS, and been associated with mortality [37]. An external validation of biomarkers and a clinical prediction model for hospital mortality in ARDS included SP-D and IL8 in various clinical settings, and suggested that these biomarkers may be useful in risk assessment for clinical trial enrolment [38].

### Coagulation and fibrinolysis

Plasminogen activator inhibitor-1 (PAI-1) is an inhibitor of fibrinolysis. Some studies have shown an increase in serum levels in patient with ARDS [18, 39, 40], and there is reported correlation with overall mortality in critically ill patients [41].

### Combinations of biomarkers

Several studies have looked into markers of epithelial and endothelial injury, coagulation and inflammation and have shown a combination of clinical predictors with combination of biomarkers were better at predicting mortality compared to either of the clinical or biomarkers alone [18, 33, 34, 42]. A panel of biomarkers was superior to clinical risk factors alone in predicting mortality in ARDS [18], as well as being useful for the diagnosis of ARDS [27]. A combination of RAGE and Ang-2 were superior to clinical diagnosis for the diagnosis of ARDS in severe trauma [43]. In severe sepsis a panel which included RAGE, SPD, Club Cell Protein 16 was useful in diagnosis of ARDS [42].

### Blood biomarkers of ARDS: Calfee group, SF

In recent years the clinical research group of Carolyn Calfee and colleagues have performed clinical studies assessing panels of blood biomarkers in ARDS. In the era of precision medicine and personalization, the Calfee group studies proceed on a more detailed characterization of the disease that may vary on individual level. They describe different ARDS subphenotypes and work on biomarker panels that may help clinicians to select patients who may benefit from different therapeutic strategies.

*Direct (epithelial)/Indirect (endothelial) Groups:* Molecular phenotyping was carried out to demonstrate 2 groups of ARDS patients: with direct (lung epithelial) damage and indirect (vascular endothelium) damage. Direct lung injury mainly caused by pneumonia and aspiration is characterised by more severe lung epithelial injury and less severe endothelial injury. For indirect lung injury, the emphasis is on endothelial or vascular injury, as opposed to epithelial damage. In direct ARDS, there are higher levels of SP-D, a marker of lung epithelial damage and there were lower levels of Ang-2, a marker of endothelial injury compared to indirect ARDS. There is evidence that lower levels of von Willebrand factor (VWF) antigen, IL6 and IL8 are present in direct lung injury [44]. With these distinct molecular phenotypes, the epithelium could be a treatment target with keratinocyte growth factor for direct ARDS, whereas the endothelium could be targeted in indirect ARDS using statins and recombinant angiopoietin 1 [44].

*Hypo/Hyper Inflammatory Groups:* Unbiased latent class analysis of clinical and biomarkers characteristics of ARDS patients demonstrated hypo-inflammatory and hyper-inflammatory groups in ARDS. These have different clinical and biological characteristics, and different responses to therapy. In the hyperinflammatory group (one third of ARDS subjects), there is a higher plasma level of inflammatory biomarkers, higher vasopressor use and lower serum bicarbonate, and higher prevalence of sepsis compared to the hypo-inflammatory group. The hyper-inflammatory group had a higher mortality and fewer ventilator-free and organ failure-free days. Eight plasma biomarkers were included: surfactant protein-D (SP-D), von Willebrand factor antigen, soluble intercellular adhesion molecule 1 (sICAM-1), IL- 6 and IL-8, soluble tumor necrosis factor receptor 1 (TNFR 1), plasminogen activator inhibitor-1 (PAI-1) and protein C [13]. More recently, it has been shown that a selection of 4 biomarkers: IL-6, interferon gamma (IFN-γ), angiopoietin 1/2 and PAI-1 could be used to cluster ARDS into two biological phenotypes with different mortality rates [45]. The stability of ARDS phenotypes has been shown over the first 3 days of enrolment in 2 clinical trials [46], and they respond differently to fluid management strategies [47].

### Septic shock biomarkers: ProCESS study

A large biomarker study of 1341 individuals enrolled in the Protocolized Care of Early Septic Shock (ProCESS) trial found that proteins associated with endothelial cell permeability and hemostasis were associated with increased mortality [48]. Angiopoietin-2, soluble fms-like tyrosine kinase 1 (sFLT-1), soluble vascular endothelial growth factor receptor (s-VEGFR), thrombomodulin (TM) and vWF were all higher in patients that died. In a sub-study of 628 patients enrolled in ProCESS, higher serum biomarkers were found in patients with adverse outcomes: including biomarkers of inflammation (IL-6, TNF, IL-10), coagulation (thrombin-antithrombin complex, D-dimer), oxidative stress (urine isoprostane) and tissue hypoxia (lactate) [49].

### Influenza ARDS: MOSAIC

The H1N1 influenza A virus is known to be associated to high morbidity and mortality. The infection can cause a severe acute respiratory failure or ARDS with multiorgan failure. The H1N1 pandemic of 2009 saw many cases of severe ARDS with refractory hypoxemia that needed the veno-venous extracorporeal membrane oxygenation as a rescue therapy [50, 51]. Recently, the interferon-inducible transmembrane (IFITM3) protein has shown in models to have a pivotal role in defending the host from pathological virus such as influenza A. In human individuals hospitalized for influenza H1N1/2009 virus it has been found elevated expression of a minor IFITM3 allele and in vitro minor CC genotype IFITM3 has reduced influenza virus restriction [52].

### Metabolomics

The analysis of lower molecular weight cell metabolites is generally performed using nuclear magnetic resonance (NMR) and mass spectrometry (MS). Metabolic changes are highly dynamic and offer insight into chemical processes occurring at any given time [53]. This makes metabolomics a useful way to detect physiological changes in real time allowing monitoring of potential environmental insults, disease progression and drug responses. These metabolite changes occur in relation to alterations in the gene and protein activity that are associated with the disease [54]. In a single study there were 4513 metabolites identified in human blood but this is an underestimate based on the individual analytical method [55]. Although many studies have been performed to assess the application of metabolomics to lung disease, progress has been slow [54].

There have been several metabolomics studies in experimental models looking at a variety of samples from exhaled breath, serum, bronchial alveolar lavage and lung tissue which find that lung injury results in a perturbation of energy and oxidative stress metabolism [54]. In contrast, there have been few clinical metabolomics studies in ARDS. Bos et al. looked into exhaled breath and analysing volatile organic compounds (VOCs) using gas-chromatography and mass spectrometry (GC-MS). Most of the candidate markers are linked to lipid peroxidation. Only octane – one of the end products of lipid peroxidation – has been validated in a temporal external validation cohort and is at the moment is most acknowledged breath biomarker for ARDS [56]. Acetaldehyde and 3-methylheptane have also been reported as predictive of ARDS and diagnostic accuracy was improved with the Lung Injury Prediction Score (LIPS) [56].

Metabolomic analysis of bronchoalveolar lavage (BAL) has been carried out using untargeted liquid chromatography mass spectrometry (LC-MS). Metabolites involved with energy metabolism such as lactate, citrate, creatine and creatinine have been shown to be associated to ARDS, and have previously been shown to be increased in plasma [57]. In addition, several guanosine network metabolites have been found to be increased in ARDS BAL fluid [54].

Langley et al. looked at extensive targeted metabolomics profiling of > 300 metabolites in two adult populations at high risk of death; patients in Community Acquired Pneumonia and Sepsis Outcome Diagnostics (CAPSOD) and patients in the Registry of Critical Illness (RoCI) [58]. Despite the phenotypic differences, most metabolites associated with mortality were upregulated.

The application of NMR-based metabolomic analysis on urine sample of pneumonia patients demonstrated the existence of different metabolomic profiles specific for bacterial infections, particularly for Streptococcus pneumoniae and *Staphylococcus aureus*. The results highlight the potential of metabolomics for the diagnosis and monitoring of the antibiotic therapy of pneumonia both in community- and hospital-acquired ones [59].

In ARDS patients higher urine H_2_O_2_ levels were associated with worse clinical outcomes, perhaps reflecting greater oxidative injury in these patients [60]; while higher urine NO levels were associated with increased survival [61]. Urinary indices of glycosaminoglycan (GAG) fragmentation, a product of degradation of the endothelial glycocalyx, individuated by mass spectrometry on urine samples in patients with septic shock or ARDS may predict acute kidney injury and in-hospital mortality [62].

### Transcriptomics – Gene expression mRNA

A recent multicohort analysis of whole blood gene expression data for ARDS by Sweeney et al. looked into 3 adult cohorts with sepsis, one paediatric cohort with acute respiratory failure and 2 datasets form adults with trauma and burns for a total of 148 cases of ARDS and 268 cases of critically ill controls. 30 genes were associated with ARDS – many of which have previously been associated with sepsis but with adjustment for the clinical severity score – none of these genes remained significant indicating that the gene expression is one of acute inflammation as opposed to lung injury [63].

Sweeney also looked separately into sepsis subtypes using data pooled from 14 bacterial sepsis transcriptomics (*n* = 700). Using cluster analysis Sweeney showed that there are 3 subtypes termed ‘inflammopathic, adaptive and coagulopathic’. Adaptive subtypes are associated with lower clinical severity and lower mortality rate and the coagulopathic subtypes are associated with higher mortality and clinical coagulopathy [64].

### MicroRNA (MiRNA)

MiRNA is a novel pathway for non-coding RNA molecules that regulate gene expression at the post-transcriptional level. It plays an important role in inflammation or apoptosis which commonly manifests in ARDS [65]. They are good candidates as disease biomarkers due to numerous factors. They can be identified in various body fluids, resistant to extreme environmental conditions, their expression changes in various disease states and change in early stages of gene expression. MiRNA can be readily measured and hence are potential therapeutic targets as each miRNA regulates the expression of many genes [66]. Most of the studies to date have been on animal models but one of the first miRNA studied in patients was miRNA-150 which has been shown to be in lower concentrations in the septic cohort; although statistical significance was not achieved [67]. Plasma levels of miRNA – 146a and miRNA155 significantly increased in patients with severe sepsis and sepsis induced ALI compared to control [68] and may be helpful in predicting mortality. A more recent study by Zhu et al. examined ARDS patients’ vs critically ill at-risk controls and identified whole blood miRNA markers in ARDS including miR-181a, miR92a. MiR-424 was shown to be a protective biomarker. Zhu concluded stating in addition to the miRNA, addition of the LIPS can improve the risk estimate of ARDS [69].

### Genetics (DNA)

Genomics in ARDS has offered relatively modest advances in understating ARDS [53]. Candidate gene studies have identified variants in more than 40 candidate genes associated with the development (or outcome) of ARDS [70]. This included genes for angiotensin-converting enzyme (ACE), IL-10, TNF and vascular endothelial growth factor (VEGF). In the first human genome-wide association study (GWAS) for ARDS susceptibility, Christie et al. identified a novel locus PPFIA1 as a replicable risk factor for ALI following major trauma, but no polymorphism had genome wide significance [71].

## Therapies

Current therapies for ARDS are summarized in Table 4. The reader is directed to excellent recent reviews that refer to modern treatment for ARDS in detail [7, 72–74]. Most advances have been through changes in mechanical ventilation methods, culminating in a 2017 International Clinical Practice Guideline for mechanical ventilation on adults with ARDS [75]. The guidelines address 6 interventions: low tidal volume and inspiratory pressure ventilation, prone positioning, high-frequency oscillatory ventilation, higher versus lower positive end-expiratory pressure, lung recruitment manoeuvres, and extracorporeal membrane oxygenation. Otherwise treatment is supportive and palliative, with no current disease-modifying therapies available. Early goal-directed therapy (EGDT) involving a 6 h resuscitation protocol of fluids, vasopressors, inotropes and red cell transfusion for septic shock did not result in better outcomes than usual care [76].

**Table 4:** Summary of therapies for acute respiratory distress syndrome

Supportive therapy	Comment
Lung protective ventilation with low tidal volume (4–8 ml/kg predicted body weight) and low inspiratory pressures (plateau pressure < 30 cmH_2_O)	Strong recommendation [75]
Higher level of PEEP^§^ in patients with moderate or severe ARDS	Conditional recommendation [75]
Lung recruitment maneuvers in patients with moderate or severe ARDS	Conditional recommendation [75]
Prone positioning for more than 12 h/die in patients with severe ARDS	Strong recommendation [75]
HFOV	Strong recommendation *against* the routine use of HFOV [75]
ECMO	Rescue therapy for refractory hypoxemia in severe ARDS. No recommendation is made, additional studies are needed [75].
Conservative fluid management strategy	It shortened the duration of assisted ventilation in large randomized trial [94, 95]
Pharmacological therapy	
Glucocorticoids	Inconclusive results on doses and duration of treatment. May provide some benefit on oxygenation, reduce inflammatory process and ventilation days. They are harmful if started 14 days after ARDS diagnosis [96].
Inhaled nitric oxide (NO)	Improves transiently oxygenation. Does not affect mortality. Higher grade of AKI [80].
Neuromuscolar blockade	Improve outcomes in patients with moderate to-severe ARDS, ensures patient–ventilator synchrony and reduces the risk of VILI [81]. Higher risk of diaphragm atrophy and ICU acquired weakness. Ongoing trial (NCT02509078).
Mesenchimal stem cells	Phase 2a clinical trials to establish safety in ARDS are in progress and two phase 1 trials did not report any serious adverse events [81].

^§^*PEEP* positive end-expiratory pressure, *ARDS* acute respiratory distress syndrome, *HFOV* high frequency oscillatory ventilation, *ECMO* extra-corporeal membrane oxygenation, *AKI* acute kidney injury, *VILI* ventilator-induced lung injury, *ICU* intensive care unit

Glucocorticoids may improve oxygenation and airway pressures in established ARDS, but have failed to demonstrate a role in preventive therapy. In patients with pneumonia, steroids may improve radiological appearances, but again does not improve mortality [77]. Trials conducted on established ARDS investigated different doses and duration of treatment, preventing generalization of the results, however analysis suggest that if steroids are started 14 days or more after the diagnosis of ARDS, they can be harmful. The combination of inhaled β_2_-agonists and glucocorticoid administered early in patients at risk of ARDS has recently shown to prevent development of ARDS and improve oxygenation but its effect on mortality has not been demonstrated [78].

Pre-hospital aspirin has been shown to reduce the incidence of ARDS, however results of ongoing trials investigating its preventive role are inconclusive and more data are needed [79]. Inhaled NO transiently improves oxygenation and long term lung function in patients who survive, but does not affect mortality and may cause renal impairment, hence it is not recommended [80]. A neuromuscular blocker, cisatracurium besilate has shown some benefit when used for early ARDS [81]. Statins [82], beta-agonists [83], non-steroidal anti-inflammatory drugs (NSAIDs), and an antioxidant (procysteine [L-2-oxo-thiazolidie-4-carboxylic acid]) have failed to show benefit for ARDS. Surfactant replacement [84], neutrophil elastase inhibitors and anticoagulation have also all failed in clinical trials.

Biologics directed against TNF have been highly successful in rheumatoid arthritis, but no benefit has been seen for the treatment of inflammatory lung diseases, including ARDS [85, 86]. With a view to lung regeneration, cellular therapies and intravenous mesenchymal stem cell therapy are in development for ARDS [87] [88]. There is significant interest in the targeted use of anti-inflammatory therapies in patients with ARDS defined by blood biomarker levels [89]. It is logical to study effects of anti-inflammatory agents in patients with hyper-inflammatory ARDS, and to tailor use of specific anti-inflammatory drugs to ARDS patients with particular biomarker profiles [90].

Despite intense investigation, no specific pharmacological treatment for ARDS has been shown to affect the mortality, even though preclinical trials in animal models have looked promising. Targeting a single pathogenetic pathway is not unlikely to be advantageous due to the complexity of the mechanisms involved.

However, the characterization of the ARDS subphenotype by blood biomarkers may help clinician to select patients who may benefit from specific therapeutic strategy and ultimately tailor the treatment on our single patient. In fact, it has been proved that a high PEEP strategy in ARDS patients affected major outcome only in the hyperinflammatory subphenotype [91]. Moreover the restrictive fluid strategy was beneficial in the same selected ARDS patients [47]. More studies are needed to further explore the benefits of different therapies based on particular ARDS biomarker profile.

## Conclusions

ARDS is a syndrome still associated with high mortality. The main treatment in order to reduce mortality relies on the correct strategic use of mechanical ventilation aimed to protect the lung by avoiding the pro-inflammatory mechanisms triggered by mechanical ventilation. The latter, however, does not represent the real treatment of ARDS since it is aimed to preserve the respiratory exchange, preserving life and allowing physicians to wait for the resolution of the underlying disease. To further reduce mortality, the therapy of ARDS should be based on the inflammatory mechanisms responsible of the lung injury, possibly taking into account the genetic difference among patients and the origin of ARDS, such as the primary or the secondary ARDS.

## Abbreviations

ACE: Angiotensin-converting enzyme
AECC: American–European Consensus Conference
AKI: Acute kidney injury
ALI: Acute Lung Injury
Ang-2: Angiopoietin-2
ARDS: Acute Respiratory Distress Syndrome
BAL: Bronchoalveolar lavage
CAPSOD: Community Acquired Pneumonia and Sepsis Outcome Diagnostics
CC16: Clara cell 16 protein
CPAP: Continuous positive airway pressure
DAD: Diffuse alveolar damage
DNA: Desossiribonucleic Acid
ECMO: Extra-corporeal membrane oxygenation
EGDT: Early goal-directed therapy
GC-MS: Gas-chromatography and mass spectrometry
GWAS: Genome-wide association study
HFOV: High frequency oscillatory ventilation
ICU: Intensive Care unit
IFITM3: Interferon-inducible transmembrane 3
IFN-γ: Interferon gamma
IL-10: Interleukin 10
IL-1RA: Interleukin 1 receptor antagonist
IL-1β: Interleukin 1β
IL-6: Interleukin 6
IL-8: Interleukin 8
KGF: Keratinocyte growth factor
KGF: Keratinocyte growth factor
KL-6: Krebs von den Lungen-6
LC-MS: Liquid chromatography mass spectrometry
LIPS: Lung Injury Prediction Score
mRNA: Micro RiboNucleic Acid
MS: Mass spectrometry
NMR: Nuclear magnetic resonance
NO: Nitric oxide
NSAIDs: Non-steroidal anti-inflammatory drugs
PAI-1: Plasminogen activator inhibitor-1
PaO_2_/FIO_2_: Oxygen arterial partial pressure/oxygen inspired fraction ratio
PEEP: Positive end-expiratory pressure
ProCESS: Protocolized Care of Early Septic Shock
RoCI: Registry of Critical Illness
sFLT-1: Soluble fms-like tyrosine kinase 1
sICAM-1: Soluble intercellular adhesion molecule 1
SP: Surfactant protein
SPB: Surfactant protein B
SPD: Surfactant protein D
sRAGE: Soluble receptor for advanced glycation end-products
s-VEGFR: Soluble vascular endothelial growth factor receptor
TM: Thrombomodulin
TNFR 1: Tumor necrosis factor receptor 1
TNFR: Transforming Growth Factor Receptor
TNF-α: Transforming Growth Factor α
VEGF: Vascular endothelial growth factor
VILI: Ventilator-induced lung injury
VOCs: Volatile organic compounds
VWF: Von Willebrand factor
